# Gangs and a global sociological imagination

**DOI:** 10.1177/1362480616659129

**Published:** 2016-08-04

**Authors:** Alistair Fraser, John M Hagedorn

**Affiliations:** University of Glasgow, UK; University of Illinois-Chicago, USA

**Keywords:** Bourdieu, comparison, C Wright Mills, gangs, globalization, reflexivity

## Abstract

Across the globe, the phenomenon of youth gangs has become an important and sensitive public issue. In this context, an increasing level of research attention has focused on the development of universalized definitions of gangs in a global context. In this article, we argue that this search for similarity has resulted in a failure to recognize and understand difference. Drawing on an alternative methodology we call a ‘global exchange’, this article suggests three concepts—homologies of habitus, vectors of difference and transnational reflexivity—that seek to re-engage the sociological imagination in the study of gangs and globalization.

Around the world, the youth gang phenomenon has become an important and sensitive public issue. In communities from Los Angeles to Rio, Capetown to London, the real and perceived threat from highly visible, street-based groups of young people has come to dominate news headlines, policy guidelines and research agendas. At the same time, the image of ‘the gang’ has become globally recognized and consumed, mediated through film, popular culture and ‘real-life’ TV series. ‘Gangs’ are depicted as an episodic phenomenon comparable across diverse geographical sites, with the US gang stereotype often operating as archetype. Mirroring this trend, academic researchers have increasingly sought to survey the global topography of gangs through positivist methodologies that seek out universal characteristics of gangs in different cultural contexts. In this article, we argue that these top–down definitions privilege a static view of gang membership that neglects the localized meanings, historical antecedents and cultural contexts of gangs. These definitions fail to capture the fluidity and contradiction inherent in gang identification, foreclose the capacity of gangs to develop into either pro-social organizations or more organized criminal entities and create an artificial sense of similarity between diverse cultural contexts. In the process, gang research has become disengaged with the broader current of sociological theory, resulting in a narrowing in the representation and analysis of diverse street-based groups.

In this article, we argue that new theoretical and methodological tools are required to understand the global gang phenomenon from the bottom–up. In making this argument, we draw on a transnational research exchange between Glasgow and Chicago which drew on comparative ethnographic observations. Grounding comparative analysis at this level reveals significant divergences in the nature, meaning and history of gang identification in these two contexts, and a corresponding difficulty with employing a common definition or response. In proposing ways to make sense of these differences, drawing particularly from the sociological thought of Mills and Bourdieu, we argue for the need to re-engage the sociological imagination in gang research through engagement with the intersecting issues of social structure, individual biography and cultural context. First, we discuss some of the principal shortcomings of current theoretical and methodological approaches to gangs in a global context, and outline the principles and practice of our ‘global exchange’ as an alternative. Following this, we introduce three concepts—homologies of habitus, vectors of difference and transnational reflexivity—which build from our shared experiences to construct a comparative theoretical framework. In the conclusion, we argue for the need for a new critical sociology of gangs in a global context, cultivating a global sociological imagination that is rooted in the history, culture and politics of distinct urban locales while recognizing the intimate connections between social structure and individual disposition. By so doing, we hope to demonstrate the potential of a global sociological imagination in criminological research more broadly.

## Gangs and globalization

The interlocking processes that have developed under the heading of ‘globalization’—namely ‘the progressive enmeshment of human communities with each other over time and […] the complex social, economic and environmental processes that stretch across their borders’ (Held, 2000: 394)—have had important ramifications for the study of gangs (Brotherton, 2007). While processes of globalization have in some cases led to convergences in lifestyles and behaviours in distal communities, these remain marked by lines of global stratification, in which social, cultural and spatial mobility is a central motif. As Bauman (2000: 2) notes, ‘[a]longside the emerging planetary dimensions of business, finance, trade and information, a “localizing”, space-fixing process is set in motion […] freedom to move […] fast becomes the main stratifying force of our late-modern or postmodern times’. Processes of socio-spatial segregation and inequality have cohered in the development of spaces of ‘advanced marginality’ in urban locales across the globe (Wacquant, 2008a). The interconnection between these areas is exhibited in the development of informal ‘grey’ economies, street justice and territorial protectionism; conditions in which the informal social order of gangs can play a functional role (Sanchez-Jankowski, 1991).

In this context, academic researchers have become increasingly sensitized to the global nature of gang activity, and have sought to survey the global landscape of gangs (Klein et al., 2001; Van Gemert et al., 2008). These efforts are epitomized most clearly in the work of the Eurogang network, a group of European and US gang researchers who have developed a set of common definitional criteria for the purposes of cross-national research, comprising ‘any durable, street-oriented youth group whose involvement in illegal activity is part of its group identity’ (Van Gemert, 2005: 148). This broad-based definition focuses on the core criteria of durability, street-orientation, youth, identity and—crucially—illegal activity. According to this definition, there are identifiable gangs or ‘troublesome youth groups’ in a range of European cities, exhibiting similar characteristics to their US counterparts. While the programme of work incorporates qualitative components, in the form of suggested city-level data and ethnographic ‘guidelines’, the comparative programme has tended towards quantitative methodologies. Latterly this definition has come to form a foundation for transnational, quantitative studies of gang membership and crime through the ISRD-2 programme of research, comprising a comparative study of youth and delinquency in 30 countries (Gatti et al., 2011; Junger-Tas et al., 2012).

While comparative research of this kind is instructive insofar as it sketches the outline of street-based groups in a global context, the deployment of a common definition through solely quantitative measures is problematic for a number of reasons. It must be remembered that the term ‘gang’ is an English word that developed to describe a particular social phenomenon in the United States and the United Kingdom. While international researchers have documented street-based criminal collaborations across a range of transnational sites (Hazen and Rodgers, 2014), the term ‘gang’ does not always easily map onto these groups. Translations of the term ‘gang’ risk misinterpretation of different cultural environments; linguistic categories evolve to describe social phenomenon as they exist within the local context. This is not simply a case of the difficulties of translation involved in cross-national research, but the more fundamental problem of the imposition of a categorization of human behaviour developed within a particular Anglo-American criminological context to cultural environments wherein these categorizations have little or no meaning. To take one example, in Mandarin Chinese there are at least four translations for the term ‘gang’, each of which has a different meaning and connotation. The most commonly used, *bangpai*^1^ (帮派), can at times have criminal connotation but at others is neutral; and can be used to describe an independent group as well as a part of a larger organization. As with many languages, moreover, there are a wide range of local dialects that draw on different cultural and linguistic roots in categorizing and labelling different aspects of social life. As Webb et al. (2011) discovered in a quantitative study of gang identification between the United States and China, these linguistic and cultural divergences can lead to fundamental problems of comparison. It is also worth noting here that there are uncomfortable convergences of geo-political and intellectual power in the construction of ‘global’ definitions of gangs, as there are in contemporary criminological knowledge more broadly (Aas, 2011). Patterns of ‘global’ research tend to emanate almost exclusively from the United States and Europe, containing inherent echoes of colonial power (Bowling, 2011; Brotherton, 2015).

As we will go on to argue, however, even in English-speaking contexts where the term has more cultural resonance—and a closer connection with lived reality might reasonably be expected—there are problems with applying a strict definition. In the United Kingdom and United States, for example, both authors studied groups that fulfilled the criteria of durability, street-orientation, youth and illegal activity. In Glasgow and Chicago, street-based groups of young men, associated with particular territories, engaging in some form of illegal activity, have been reported for over a century. We contend, however, that street-based groups in Glasgow and Chicago have followed radically divergent trajectories, and consequently involve fundamental differences in their meaning, form and context. As a result, a quantitative survey seeking to compare these sites through a common definition would simply not be measuring a phenomenon sufficiently similar as to be useful. These differences, we argue, have resulted from the different social, structural and spatial development of the cities themselves, and the national contexts in which they are situated. In Glasgow, there is ‘durability’ insofar as certain gang names have been reported for over a century. However, this is not a self-sustaining criminal organization but a hand-me-down identity that is refitted and recast by successive generations—and that cannot be understood outside of the broader economic and social currents of the city of Glasgow (Fraser, 2015). In Chicago, by contrast, durability is constituted by the institutionalization of street gangs in local drug economies, in response to severe social and economic marginalization, yet in some cases evolved into social movements and political parties (Hagedorn, 2015). In both cases, it is impossible to make sense of the meanings of gangs without a careful appreciation of the history, sociology and politics of the broader urban context.

One consequence of an over-reliance on quantitative comparison, or indeed a qualitative approach with a closed definition, is the relegation of the importance of sociological theory in making sense of gangs in a global context. Where once issues of history, culture and class were at the heart of theories of gangs, latterly issues of crime, policing and risk management have come to dominate. As a result, gangs are too often reduced to a fixed, static and ‘monolithic entity, with a single-mindedness of purpose and outlook’ (Venkatesh, 2003: 8); which is presented as a universal social form. In the context of an increasingly divisive policy environment in both the USA and the UK, in which gangs are often a convenient scapegoat for a broader set of social anxieties, there is a danger that academic research can reify the gang phenomenon as an objective, criminal entity, offering justification for increasingly punitive policy responses. Rather than seeking to refine definitions, we argue that there is a need to reconnect the study of gangs with the social, cultural and biographical processes through which gang identification is constituted—a project that is undercut by excessive prescriptiveness in relation to definitions.^2^ In making this case, we draw particularly from the rich transatlantic sociological theory of Pierre Bourdieu (1984, 1990, 2005) and C Wright Mills (1959). Though there are significant differences in their approaches, both evidence a disdain for ‘grand theory’ and ‘abstract empiricism’—approaches to the social world that fail signally to connect with what they saw as the principal foundations of a sociological understanding of the world—while seeking to unite structure, culture and agency in understanding the social world (Burawoy, 2010).^3^ In an effort to reconnect the study of gangs with a sociological imagination, we seek to develop new methodological lenses with which to recognize and understand difference while remaining grounded in empirical realities.

## Gangs and global exchange

The reconfigurations of social life wrought by processes of globalization have had far-reaching consequences across the social sciences, engendering a range of novel articulations of theory and method. Specifically, globalization has posed important methodological challenges to criminology as a discipline, as the national borders of crime, security and justice are increasingly traversed and transgressed. These developments call into question the ‘methodological nationalism’ (Beck and Beck-Gernsheim, 2009) that has traditionally characterized criminology, necessitating new forms of comparative, transnational and globally informed research. As Bowling (2011: 363, emphases in original) notes of these developments:

global criminology aspires to bring together transnational and comparative research from all regions of the world to build a globally inclusive and cosmopolitan discipline […] *Transnational criminology* goes beyond comparative analysis to explore problems that do not belong exclusively in one place or another and can therefore be understood by analysing *linkages between places*.

In developing such transnational links, it is necessary to move beyond a top–down approach to definition and learn from grounded comparisons that are situated within a broader structural context. Rather than starting with deductive reasoning, for example, Wacquant’s (2008a: 9) ‘comparative sociology of urban marginality’ between Chicago and Paris seeks to compare geographically disparate sites inductively. As Wacquant (2008a: 9) notes, first-hand observation is ‘an indispensable tool, first to pierce the screen of discourses whirling around these territories of urban perdition […] and secondly to capture the lived relations and meanings that are constitutive of the everyday reality of the marginal city-dweller’. This form of comparative ethnography, however, is exceptionally rare. Long-term engagement with diverse urban milieu requires a bilingual cultural sensitivity and scholarly commitment that is as demanding as it is time-consuming. Burawoy’s (1991, 2000) collaborative ethnographic projects offer an alternative that is rooted in efforts to comprehend the global ‘forces, connections and imaginations’ in which increasingly interconnected, yet disparate, social realities can be grasped through in-depth observation.

Drawing inspiration from these insights, between 2009 and 2010 the authors conducted a transnational exchange between our respective fieldsites, with the ‘home’ researcher operating as a gatekeeper, guide and critical friend during the field-visit. The purpose was to physically experience a different fieldsite, and be confronted bodily with the similarities and differences with the ‘home’ research site. The beauty of the exchange is its simplicity and efficiency: the hard-won access of the other researcher is shared and collectivized, allowing the visiting scholar a sharp, penetrating insight into a social world that may diverge considerably from their own. The idea came about partly by accident, and partly by design. As a doctoral student, Fraser spent three months in Chicago (September–November 2009), carrying out informal interviews and observations with a wide range of community activists, young people, gang members, police officers and academics. Despite starting from a presumption of similarity, he learned about the vast differences between what are referred to as ‘gangs’ in Chicago and Glasgow, demonstrating unequivocally the sharp divergences in the gang phenomenon in apparently similar contexts. Despite its relative brevity, the experience felt akin to Nelken’s (2000: 147) depiction of ‘long-stay’ researchers ‘living there’, who are ‘engaged in a process of being slowly re-socialized […] [and] may doubt whether they ever really understood their own culture of origin’. Sharing a desire to explore further the differing trajectories of gangs in the two cities, Hagedorn spent a month in Glasgow in December 2010, travelling to fieldsites, reading and talking to scholars and community leaders about Glasgow’s history. For him, the experience was closer to Nelken’s (2000) depiction of ‘researching there’ rather than ‘living there’. Time spent at a site changes perceptions; the sights, sounds and smells of a place sharpen the comparative senses in ways texts can never suffice. It prompted the asking of new questions that may differ or seem naïve if asked by an insider. While gangs in Glasgow and Chicago seemed to have begun in similar ways and both have persisted for more than a hundred years, the experiment was intended to understand how these (not so small) differences in gang organization and activities can be understood in radically different contexts (Burawoy, 2009: 202).^4^

Since this initial physical exchange, both authors have completed monographs on the gang phenomenon in their respective cities; the former premised on the accumulation of 10 years of research and scholarship on the Glasgow gang phenomenon, the latter on 20 years of Chicago-based research (Fraser, 2015; Hagedorn, 2015). In what follows, we reflect on the methodological and theoretical implications of this experiment in qualitative comparative research, and its implications for global and comparative gang research. While fundamentally rooted in our fieldwork experiences, our intention is to ‘extend out’ beyond Glasgow and Chicago to create dialogue with scholars of gangs around the world (see Hazen and Rodgers, 2014). Based on the comparative insights that flowed from the initial exchange, and drawing on these historical analyses of our respective cities, we suggest three concepts—homologies of habitus, vectors of difference and transnational reflexivity—that seek to cultivate a global sociological imagination in relation to gangs. These concepts seek to shuttle between the levels of everyday experience and disposition, urban history and cultural context to compose an analysis that is both grounded and comparative. In the words of Mills (1959: 211), this approach ‘consists of the capacity to shift from one perspective to another, and in the process to build up an adequate view of the total society and its components’.

## Homologies of habitus

In Bourdieu’s terms, ‘habitus’ refers to the set of durable character dispositions—habits—that all individuals possess (Bourdieu, 2005; Bourdieu and Wacquant, 1992). These dispositions are both intellectual and physical and frequently operate at an unconscious, or *pre*conscious level, giving the feeling of being instinctive. Daily interactions are structured by our habitual range of responses within a specific ‘field’ of action—be it street-based, bureaucratic or academic (Shammas and Sandberg, 2016)—whose logic, rules and forms of ‘capital’ are deeply embedded in our daily routines. Bourdieu likens this to a ‘feel for the game’—an instinctive response to learned rules (Bourdieu and Wacquant, 1992: 128). Habitus is, therefore, a concept that draws attention to the generative historical structures of inequality—for example of class, gender and ethnicity—through which individual dispositions are patterned, while recognizing the role of individual decision making and agency within this broader context. Bourdieu (1984) most famously elaborated these ideas in *Distinction*, demonstrating that tastes, beliefs and politics are fiercely patterned by the matrix of class position at an objective level, while simultaneously differentiated and unpredictable at the subjective level. As Wacquant (2008b: 267) summarizes:

These unconscious schemata are acquired through lasting exposure to particular social conditions and conditionings, via the internalization of external constraints and possibilities. This means that they are shared by people subjected to similar experiences even as each person has a unique individual variant of the common matrix (this is why individuals of like nationality, class, gender, etc., spontaneously feel ‘at home’ with one another).

In both Glasgow and Chicago, street-socialized youth exhibited a deep-seated form of territorial place attachment that reflected a limited and limiting spatial immobility that might be characterized as a form of ‘street habitus’ (Fraser, 2013a). This comparable form of territorial identification in the midst of severely disadvantaged communities was evident across both fieldsites; as was the devastating impacts of social and economic change on the communities visited. Globalized processes of marginalization—gentrification, displacement and precariousness—were therefore implicated in the habits and traits in young people’s everyday lives. While there were marked differences, we suggest that there are homologies that revolve around persistent inequality, socio-spatial segregation and territorial identity, representing adaptive responses to global economic forces. Approaching the study of gangs in this way allows for an equivalence to be drawn with the structured routines and dispositions within other social fields. As Burawoy (2010) has recently argued, both Mills and Bourdieu argued for the necessity of understanding the stratified way in which power is reproduced across various spheres of cultural production, as well as the complex bureaucratic mechanisms that disguise patterns of social reproduction and symbolic violence. As recent work in Bourdieusian criminology has demonstrated (Shammas and Sandberg, 2016), there are comparable patterns of status contest, trading of cultural capital and mystification involved in a range of criminal justice contexts. Approaching gang identification through the lens of street habitus allows a proper appreciation of the role of structural and symbolic violence in the concatenation of street-based identities.

Beyond our fieldsites, research carried out on what has been termed ‘inner-city street culture’ demonstrates that similar traits have been documented in other urban contexts. Bourgois, basing his findings on extended participant observation in *El Barrio* in Brooklyn, New York, distinguishes the set of dispositional qualities that obtain in this community. For Bourgois (1995: 8), this street culture has ‘emerged in opposition to exclusion from mainstream society’. Anderson (1999), in *Code of the Street* identifies a similar set of ‘street’ dispositions in low-income communities in Philadelphia. Sandberg’s (2008) research in Oslo documents an embodied, streetwise disposition among street-based drug-dealers—in which bodily capital, language and street smarts are employed to navigate violent social terrain—that represents the internalization of the experience of marginality and the strategic employment of forms of available capital. In an evocative account of the life trajectories of three brothers in and out of gangs in Springfield, Massachusetts, Black (2009) underscores the depth, complexity and contradictions in their daily lives, and the structuring role of everyday marginalization in these experiences. Drawing explicitly on Mills, Black (2009: 357) makes an impassioned case for a sociological imagination in relation to gangs, via analysis of ‘neoliberal economic capitalism, through the social institutions that organise it, to the immediate milieus of marginalised urban minority communities’. These shared traits between diverse geographical locales represent adaptive responses to convergent economic trends (Richardson and Skott-Myhre, 2012), representing a complex street-based reality that is not easily approximated through quantitative survey (Brotherton, 2015; Hobbs, 1997; Katz and Jackson-Jacobs, 2004). As Jock Young (2004: 25–26) argues, while certain phenomena are capable of definition, ‘there are many others that are blurred […] because it is their nature to be blurred’.

As such, the street-based dispositions of young people in Glasgow and Chicago must be understood as a response to the structural violence that has deeply embedded inequality and disadvantage in both cities. In this context, it is important to note the historical parallels between the two cities—as Mills (1959: 215) notes, ‘[s]ome knowledge of world history is indispensable’. In the late 19th and early 20th centuries, Glasgow was the ‘Second City’ of the British Empire; Chicago, the ‘Second City’ of the United States. Both experienced huge increases in population to become cities of more than a million inhabitants, as well as mass migration and industrialization: in Glasgow, from Ireland, Europe and the Scottish Highlands (Maver, 2000); in Chicago from Ireland, Italy, Poland and the Southern United States (Burgess, 1961 [1925]). In 1914, Glasgow produced three-quarters of the ships for the British Empire, as well as half of the locomotives (Mitchell, 2005), while Chicago was the railroad hub of the USA and provided the country with meat and steel through its central railways (Duis, 1998). During this period, both Glasgow and Chicago experienced rapid urbanization, increasing population densities and territorial gang conflict.^5^ Since this period, despite divergent trajectories, both cities have experienced persistent extremes of poverty and marginalization. In Glasgow, for example, despite successive waves of regeneration and redevelopment, these improvements have not been felt equally. To this day, some 40 per cent of Glaswegians live below the poverty line (Dorling and Pritchard, 2010).

While young people’s habits and traits were deeply rooted, however, there was evidence in both settings of internal contradiction and alteration. Across both fieldsites, both authors witnessed the ways in which gang identities waxed and waned over the life-course (Anderson, 1999; Brenneman, 2012), but also the processes through which reputations became difficult to shake beyond the period of ‘youth’ encapsulated in most common definitions. In Glasgow, one participant described the way in which her dad’s friends referred back to his youthful reputation; similarly, a police officer described an incident whereby a man in his 30s was stabbed in a cinema by a man he had fought against in his teens. In Chicago, several young adults were struggling between streetlife and ‘going straight’, while others exhibited nostalgia for past days involved in streetlife:

The afternoon was spent, nostalgically, in the home of an ex-gang member; reliving days gone by via a video-cassette of his 69th birthday party. Here are the movers and shakers of Westside Chicago in the 1960s, still sharp, still slick, still hip; dancing, and chatting, and laughing in a club. Now he is getting on in years, there is a tangible sense that he wants—and needs—this approbation to be remembered. His house is festooned with pictures of the glory days—the razor-sharp suits, the people, the still-shot in the boxing ring.

(Fieldnote, AF, October 2009)

There were therefore components of habitus that were similar between Glasgow and Chicago: historically embedded and age graded yet at times riven with internal conflict (Venkatesh, 2003). The similarities in street-based dispositions, in this sense, were deeply rooted in the economic and social history of their respective cities, and were not confined to defined age groups. Rather than representing a universal social phenomenon, we suggest that these are best represented as ‘homologies’ to denote the comparability of traits emerging from similar structural conditions, while recognizing their specificity to local contexts. As will be explored below, however, there was at least as much that separated street-based youth in these two contexts than united them. It is these ‘homologies of habitus’—not rigid but flexible, recognizing the interweaving of history, biography and culture—that allow us to recognize gangs in both Glasgow and Chicago as similar, while gang behaviours markedly differ. As we discovered, such convergences are always mediated through the particular cultural and social context of different cities. They constitute a patterned response to structured inertia but the emergent forms of prestige and capital are far from unique.

## Vectors of difference: A tale of two (second) cities

Glasgow seemed to me a place out of time, more Chicago School than Chicago itself. It appeared so stable. Kids fought for their neighborhood and told me it wasn’t for drugs or money. I could see why Alistair invoked Thrasher even as I was questioning the old master. I’m not sure I accepted as accurate my one month snapshot of Europe’s leading gang city. But I surely wasn’t in Lawndale anymore.

(Fieldnote, JMH, January 2011)

Our observations demonstrated significant divergences in the nature, meaning and form of gang identification between the two fieldsites. In Glasgow, gang identification was predominantly street based, youthful, territorial, usually representing a single neighbourhood or street and seldom evolving into more organized, economically motivated criminal organizations. Gang formations during different generations have reflected the changing economic and social circumstances of the city, but nonetheless forming an ongoing core that represents a place-based identity above an economic motivation (Fraser, 2015). In Chicago, by contrast, gangs play a major role in the ‘perverse connections’ (Castells, 2000) of the international drug economy; incorporating adults, multi-neighbourhood alliances and financial imperatives. While also rooted in place, gangs have evolved to play an organizing role in politics and illegal markets (Hagedorn, 2015). In order to understand how the differences in street-based dispositions unfold, we argue that there is a need to ground understanding within the context of the history, politics and culture of specific urban locales—seeking to excavate a ‘history from below’ (Brotherton, 2015) through which the divergences in gang formations can be explained. In what follows, we trace the historical trajectories of gangs in Glasgow and Chicago, pointing to what we term ‘vectors of difference’ around which these differences cohere. Such deeply cast historical trajectories, we argue, are central to a global sociological imagination, yet which tend to be evacuated in the use of common definitions.

### Fields of crime and justice

The air was bright and fresh. We sat opposite Douglas Park, Lawndale, on milk-crates; people passed by and shot the breeze. But dark clouds were forming—not in the air but on the stoop. John’s friend’s son had been robbed and shot in the back a few weeks previous—dealing drugs on the corner, he had been taken for all he had. There had been retaliatory shootings, and a war was brewing. The man was defiant, staunch and vicious in his talk of vengeance. A slight against his family was a slight against him: no quarter would be given. The same man, frankly, was disbelieving when I told him the way things were in Glasgow: youth gangs didn’t have guns, didn’t deal drugs, didn’t really make money. In stark terms, the similarities I’d thought were there were blown out of the water.

(Fieldnote, AF, October 2009)

While Glasgow-based fieldwork revealed gang identification to be a largely social phenomenon, reflecting longstanding friendships based on area coupled with embedded territorial boundaries and competitive forms of street-based masculinity (Fraser, 2015), experiences in Chicago suggested an economic motivation that was substantially different. This differing raison d’etre between street-based groups in Glasgow and Chicago was underscored time and again during interviews and observations. Unlike in Chicago, illicit opportunity structures for young people are not well developed in Glasgow—youth gangs have seldom evolved collectively into more organized groups through drug sales, racketeering or organized crime (Fraser, 2015). Rather, through a relatively closed loop of street-based age hierarchies—reflecting the persistence of inequality in Glasgow’s communities—gang identification becomes a temporary route to status and distinction that is consistent with the logic of local models of masculinity.^6^ In Chicago, by contrast, there is a complex and sophisticated web of relations between street gangs and organized crime (Venkatesh, 2008). While Glasgow’s gangs have institutionalized over a period of a hundred years, they have remained largely neighbourhood based. No lasting alliances or multi-neighbourhood gangs have emerged in sharp contrast with Chicago (Hagedorn, 2015).

Part of the explanation for this difference can be found in the role of the state (Wacquant, 2008a). Despite considerable changes over time, Glasgow has maintained a robust police force, and continues to have one of the highest ratios of police officers to citizens among world cities (Damer, 1990); as well as maintaining a welfarist ethos in criminal justice (McAra, 2008). The picture is radically different in Chicago, where the leading history of the Chicago Police is entitled “To Serve and Collect” (Lindberg, 1998). Chicago’s Mafia, the ‘Outfit’, institutionalized in Chicago after the prohibition era beer wars (Eghigian, 2006), and achieved economic success through corruption of police and politicians. When street gangs took over retail drug markets, they did not inherit the protection of senior police on the Outfit’s payroll but instead began the systematic bribery of individual police and units. A recent report on police corruption in Chicago describes the change in corruption practices as ‘from top down to bottom up’ (Hagedorn et al., 2014).^7^

Another important factor is the role of prison. Scotland imprisons approximately 8000 inmates (Scottish Government, 2012b), while the state of Illinois, with double the population of Scotland, has 49,000 inmates, more than six times the number of prisoners.^8^ The ‘deadly symbiosis’ between prison and ghetto in Chicago (Wacquant, 2001) has played a critical role in institutionalizing gang structures. In the 1970s, many gang leaders were incarcerated, leading to the creation of prison-led gang coalitions. These coalitions were organized to decrease violence at first inside the prisons and then aimed to control it on the streets; requiring local gangs to organize, to have structure, adhere to various laws and rules of behaviour and enforced neighbourhood market boundaries. While decreased violence was its own reward, reducing violence was also of benefit to illegal businesses. In this sense, Chicago’s gangs became political players to some degree and were conscious social actors (Sassen, 2006), a label that does not easily fit the Glasgow gang phenomenon.

### The conservation of violence

In 2011, Chicago saw 433 homicides, with a rate of approximately 16 homicides per 100,000 population (Chicago Police Department, 2011). For the period 2010–2011, Glasgow—reputed as the ‘murder capital of Europe’ (McKay, 2006)—experienced 27 homicides, at a rate of approximately 4.5 per 100,000 (Scottish Government, 2012a). In this period in Scotland, the majority (63 per cent) of homicides were carried out with a bladed instrument, while only a tiny fraction (2 per cent) involved a firearm (Scottish Government, 2011). In Chicago, conversely, shooting accounted for 83.4 per cent of all homicides, while stabbing constituted only 6.7 per cent (Chicago Police Department, 2011: 22). In both contexts, young males make up the highest proportion of both victims and offenders. In Scotland, homicide statistics are not routinely reported by ethnicity, but it is likely that both victims and offenders are ethnically white. In Chicago, approximately 75 per cent of homicide victims and offenders are African American, a percentage vastly disproportionate to the ethnic make-up of the city (Chicago Police Department, 2011).^9^

In making sense of these divergences, Bourdieu’s (1998) ‘law on the conservation of violence’ is particularly apposite (see also Bourgois, 2001). Bourdieu (1998: 40) equates the strength and weight of different configurations of structural violence to that of individual violence: ‘structural violence exerted by the financial markets […] is matched sooner or later in the form of suicides, crime and delinquency, drug addiction, alcoholism, a whole host of minor and major everyday acts of violence’. According to this law, the street violence of gangs will accord with the weight of symbolic or structural violence within different urban contexts. While both cities have suffered crushing declines in forms of industrial manufacturing that once breathed life into their urban heartlands, Chicago has been more successful in recreating as a tourist and fiscal centre; but this has come at great cost. While both have experienced the global processes of neoliberalism, urban exclusion and territorial stigma that Wacquant (2008a) terms ‘advanced marginality’ (see also Gray and Mooney, 2011), these processes have been both more advanced and more marginalizing in Chicago. In part, these differences have been driven by the divergent patterning of state formation, welfare policy and criminal justice in the United States and Scotland.

The differences in structural violence between Glasgow and Chicago are, however, most evident in relation to race, ethnicity and criminalization. Although Scotland has experienced several waves of immigration—predominantly different groups of Irish, Jewish, Italian, Chinese, East European and Indian subcontinent immigrants (Croall and Frondigoun, 2010: 112–113)—the country remains an overwhelmingly ethnically homogenous nation, with approximately 4 per cent of the population categorized as belonging to black and minority ethnic (BME) communities; though this increases to 12 per cent in Glasgow (Haria, 2014). To date, statistics indicate a relatively proportionate representation of minority ethnic groups in the Scottish criminal justice system, and that ‘race’ and ethnicity play a less significant role in victimization in Scotland than in other jurisdictions (Croall and Frondigoun, 2010). Fraser (2013b), for example, draws on Les Back’s notion of ‘neighbourhood nationalism’ to describe the situation among young people in the Langview community, in which differences based on ‘race’ or ethnicity are subordinated to a collective loyalty to a geographical area.

In contrast, Chicago’s history is one of racialized conflict and discrimination. During the First World War, acute labour shortages were addressed by recruiting thousands of African American workers from the South. Housing policies created ethnic segregations, and at the end of the war, rather than solidarity, territorial racial tensions escalated. As Glasgow saw a class-based solidarity in 1919,^10^ Chicago experienced a race riot that claimed 43 lives. A Race Relations Commission, which was co-chaired by a prominent member of the Chicago School, Robert Park, stated that the riot would have ended after one day except for the provocations of the white social athletic clubs.^11^ Aside from underlining and reinforcing racist divisions in Chicago, the race riots would result in a pattern of hyper-segregation that has marked Chicago ever since. Hirsch (1983) tells the story of a 1920s–1960s era of ‘hidden violence’ when white gangs, supported by police, kept the black community segregated. Between 1940 and 1960 Chicago’s African American population tripled and could no longer be spatially contained (Hirsch, 1983). The city council decided to build massive high-rise housing in all black neighbourhoods in order to keep African Americans forcibly segregated. Chicago’s gangs entrenched within the projects that became defensible spaces and captive drug markets (Venkatesh, 2008).

## Gangs and transnational reflexivity

We carry around lenses that are so much a part of us that we don’t notice we have them, yet as social scientists our task is to bring those lenses to consciousness, compare one with another, and to develop from them other more detachable lenses, which we call social theory, so that we can get on with the business of studying the social world.

(Burawoy, 2009: 205)

One consequence of the operation of habitus, field and capital is the phenomenon that Bourdieu (1990: 20) terms ‘doxa’—namely the narrowing of one’s social universe to the outer limits of the field. As we discovered, academic researchers are not themselves immune to the operations of this form of narrowing. While traditional gang research tends towards sedentarism, with research carried out within researchers’ country of origin, we found that with sedentarism comes a set of institutional, cultural and national boundaries that place limits on understanding experiences beyond that country. In fact, while the concept of ‘global exchange’ was relatively straightforward, the experience itself was challenging in the extreme. We were wrenched out of comfort zones and thrust into an alien social world, in which our points of reference were out-of-sync. As Kenway and Fahey (2009: 28) note: ‘the place and movement of the researcher’s body and thought’ represent a central pivot in constructions of knowledge. It quickly became clear that the comparing of gangs in Chicago and Glasgow was the equivalent, in Wacquant’s terms, of comparing ‘heavyweights to flyweights’ (Wacquant, 2008a: 150). Our experiences suggest a need to engage in transnational research with honesty and humility.

Both Bourdieu and Mills advocated for reflexivity in social research (Burawoy, 2010). In a celebrated postscript, Mills delineates the role that the individual researcher plays in the process of knowledge production. Mills (1959: 216) argues that in order to perform sociology as a craft, researchers must ‘learn to use […] life experience in […] intellectual work: continually to examine and interpret it’. While reflexivity of the researcher’s race, class and gender has become increasingly recognized within the field of criminology (Lumsden and Winter, 2014), Bourdieu’s notion goes further, in seeking ‘a reflexive return on the sociologist and on his/her universe of production’ (Wacquant, 1989: 33). For Bourdieu, this form of reflexive analysis is intended to construct the gap between the ‘logic of practice’, in this case the sedimented forms of research practice, and the ‘logic of theory’, namely the development of explanatory conceptual frameworks, as an object of empirical scrutiny (Bourdieu and Wacquant, 1992; Maton, 2003). Bourdieu and Wacquant (1992: 36) summarize as follows:

First, its primary target is not the individual analyst but the social and intellectual unconscious embedded in analytic tools and operations; second, it must be a collective enterprise rather than the burden of the lone academic; and, third, it seeks not to assault but to buttress the epistemological security of sociology.

When we each travelled to one another’s fieldsites, we experienced these new street-based environments through the lens of a pre-existing, doxic habitus; that is, through an embedded optic that was developed within a specific cultural context (Sheptycki and Wardak, 2005). Both of us had been engaged in prolonged qualitative fieldwork in our respective cities, and had developed corresponding theoretical lenses that ‘fitted’ with that particular urban environment. Fraser (2013a, 2015) drew from a historical and cultural perspective rooted in Thrasher (1963 [1927]) and the post-industrial context of Glasgow, while Hagedorn’s (1998, 2008) approach had moved from applying Wilson’s (1987) ‘underclass’ theories to Castells’ (2004) information age city-centred, global perspective in an effort to comprehend the changing dynamics of gangs in a global context. These perspectives, crucially, were deeply embedded—both found it difficult to think outside of their boundaries. For example, on hearing repeated, sharp sounds in the Chicago fieldsite, Fraser asked if these were firecrackers—the notion that gunfire could be heard at almost any time of day or night in the community was far outside his Glasgow experience. Hagedorn, conversely, remained unconvinced that the pattern of racialized discrimination and violence that was so embedded in Chicago could not be replicated in Glasgow.

As Bourdieu (2005: 46, emphasis in original) points out in relation to such contradictions, ‘in all the cases where dispositions encounter conditions (including fields) different from those in which they were constructed and assembled, there is a *dialectical confrontation* between habitus, as structured structure, and objective structures’. Habitus does not automatically accede to the new conditions of the field—as the saying goes, ‘old habits die hard’. Both researchers came to see their own fieldsites with fresh eyes, helping to cultivate an awareness of the social and cultural roots of our own perspectives. Fraser, for example, learned about the ‘silences’ in his own fieldwork—the role of race, politics, organized crime and corruption—and was prompted to interrogate the institutional and individual biases that left these questions unasked. As Sallaz (2009: 7) notes:

The method of comparative ethnography is in this sense not only productive but prophylactic, insofar as it serves as a safeguard against importing into a research study one’s own common sense assumptions about the social world—a particularly acute danger when one’s purview is confined to but a single case.

The fissures and disjunctures experienced—and the debates which ensued—brought to the fore new concepts that acted as foundations for the building of new theory. Our shared experience created a discursive space in which the similarities and differences between our fieldsites could be explored. While remaining fundamentally grounded in the two fieldsites of Glasgow and Chicago, we hope that this inductive method of theory building of these concepts might contribute to future global, comparative or collaborative gang research—instigating a form of transnational reflexivity that is premised on recognizing and understanding difference rather than attempting to impose similarity.

## Conclusion: Towards a global sociological imagination

The facts of contemporary history are also facts about the success and failure of individual men and women. When a society is industrialised, the peasant becomes a worker; the feudal lord a businessman […] Neither the life of an individual nor the history of a society can be understood without understanding both.

(Mills, 1959: 3)

Mills’ delineation of the sociological imagination, over half a century ago, called for the cultivation of an approach that united history, biography and culture in making sense of complex social phenomena. In the context of globalization, scholars have been challenged to incorporate an additional layer—‘the global’—into this trinity. Within the field of gang research, however, rather than seeking out sociological understandings that engage with these structuring forces for individual dispositions, rather gang research can be said to have ‘wither[ed] under the censorship of power’ (Bourdieu, 1998: 38) as studies have tended towards a narrow, criminalizing approach to definition. In the process, theoretical accounts of gangs have become disengaged from the critical relationships that pattern the nature and form of gangs in the contemporary era (Katz and Jackson-Jacobs, 2004). In this article, we have argued for the need to re-engage the sociological imagination in understanding gangs in a global and comparative context. Several conclusions flow from our observations.

First, efforts to construct and apply top–down definitions of gangs should be treated with extreme caution. Though our exchange focused on traditional sites for gang research, in the United States and the United Kingdom, significant differences in the nature and meaning of the term were uncovered. In Glasgow, the term ‘gang’ referred to territorial youth groups that were rooted in specific communities and did not necessarily involve crime or violence; in Chicago, the term was used to describe large-scale, multi-neighbourhood adult alliances that were institutionalized in some communities. If the term represents such divergence in apparently similar cultural contexts, we suggest that there is a significant margin for error in seeking to translate this concept to other environments. We argue that researchers should be more concerned with recognizing and understanding variability in the forms and activities of gangs in different cities. Critical gang scholars have, for example, recently traced the diffusion of prison gangs to the streets (Weide, 2015) and the formation of gangs’ context of civil war in Honduras (Levenson-Estrada, 2013); as well as documenting the transformation of gangs into pro-social forms in New York (Brotherton and Barrios, 2004), organized crime in Rio de Janeiro (Dowdney, 2007) and policing units in Nigeria (Hagedorn, 2008). These studies reflect the profusion of new international studies, particularly in the global South, which have revealed gangs’ capacities to change outside the bounds of static definitional criteria. This narrowing of complexity into formal categories of similarity is also in evidence more broadly in the field of criminology, and we argue that other areas too—punishment, legal systems and policing among them—would benefit from a greater attentiveness to the relationships between history, biography and culture.

Second, we suggest a re-examination of the building blocks of criminological theories of gangs. We have drawn on the sociological insights offered by the ‘thinking tools’ of Mills and Bourdieu to explain place-based differences in gangs, as well as the relation between structural context and individual disposition. ‘Homologies of habitus’ is suggested as a replacement for subcultural theories as a way to mark similarity between gang members in different cities, while exposing differences. ‘Vectors of difference’ represents a way to explain how the history of Chicago and Glasgow manifests itself in gangs that are familiar yet possess very different characteristics. Bourdieu’s ‘law of the conservation of violence’ helps us to understand gangs as social actors, but whose actions are shaped by the historic social structures in different cities. In this way we explain racialized differences in levels of violence between Chicago and Glasgow. While comparison of other urban contexts may bear differing variables—militarism, state response and media depictions among them (Hazen and Rodgers, 2014)—our intention is to begin a conversation around the levers of change and difference in urban contexts around the world.

Third, in elucidating these ‘vectors of difference’, we report on our methodology of ‘global exchange’ as a means of capturing this diversity. While this approach is nothing new or unique in and of itself, our time in Glasgow and Chicago allowed us to view our own cities through different lenses and discover patterns of behaviour that had been hidden to us ‘like water for fish’ (Lorber, 1994). In excavating an urban genealogy of space, politics and culture, we have argued that the city operates as a lens through which to understand how broader changes are refracted and articulated, on both the group and individual level. Gangs in Glasgow and Chicago remain rooted to fundamentally local processes, flourishing in the ‘*space of places*’ (Castells, 2000: 408–409, emphasis in original), though processes of globalization had altered these local processes in unique ways. In making sense of similarity and difference between these diverse contexts, we have argued for the importance of reflexivity in recognizing the cultural and national biases in constructing social theory. We join with Mills’ (1959: 225–226) celebrated ‘sociological imagination’ in calling for a reflexive connection of history, biography and culture in gang research that encompasses ‘a fully comparative understanding of the social structures that appeared and that do now exist in human history’.

Finally, Bourdieu and Mills were both, in different ways, inured to the idea of the promise and task of sociology to bring about social change (Burawoy, 2010). For both, through the cautious development of carefully honed analytic tools and methods, it was possible to gain sufficient knowledge of the structural contexts in which we live to break out of them. While conceptual clarity is important, definitional strait-jacketing results in a view of social life and cultural change that fails to fully recognize the potential for sociology to participate in social change. The narrowing of the definition of the term ‘gang’ to durable, youthful, street-based, illegal activity negates the possibility for understanding the ways in which street-based groups emerge and sustain in relation to structural inequality, socio-spatial marginality and economic precariousness; or indeed the ways in which street-based groups can exhibit pro-social traits, conflicting identities and change over time (Coughlin and Venkatesh, 2003; Dowdney, 2007; Sanchez-Jankowski, 2003). A global sociological imagination of gangs—and of criminology more broadly—is one that seeks to understand the complexities of social structures and everyday life in order to seek out lines of social justice, be it at individual, city or international level. This is, to paraphrase Mills, its task and its promise.

## Notes

1. ‘Youth criminal groups’ (青少年犯罪团伙); ‘street-corner youth’ (街角青年); ‘school gang’ (校园帮派); and ‘youth-based organizations with the character of black society’ (青少年黑社會性質組織). For further discussion, see Wang (2013).

2. We use the term ‘gang’ in this article to refer to ‘alienated groups socialized by the streets or prisons not conventional institutions’ (Hagedorn, 2008: 31)—a broad-based definition that recognizes the role of both social structure and street culture in understanding the complexities of gang identity.

3. Burawoy (2010: 1), indeed, makes the case that ‘Mills is the American Bourdieu’.

4. Burawoy’s (2009: 49) ‘extended case method’ seeks to concatenate both objective and subjective layers of analysis, ‘tracing the source of small differences to external forces’ through ‘inductive generalization […] to seek out common patterns among diverse cases, so that context can be discounted’. Rather than stressing the *extra*local, however, following Wacquant (2008a) we privilege the *urban* whilst recognizing the shaping and structuring role played by broad-based global forces.

5. During this period, Glasgow was in fact labelled as the ‘Scottish Chicago’ (Davies, 2013).

6. Although, in one community that Hagedorn visited, he was informed that some fathers, who were territorial gang members in previous years, were running gambling and drug enterprises with their sons and current gang members doing the street-level distribution. The evidence for these associations remains underdeveloped, but there is little to suggest large-scale collaborations oriented around drug sales on the scale of Chicago’s gang scene.

7. On top of police corruption has been a pattern of abuse by police that has fed hostility on the streets. Jon Burge, a police commander who learned torture tactics in Vietnam, systematically applied them to black gang members, and at least 119 gang members have been confirmed to have been brutally tortured by Burge. The #BlackLivesMatter movement, which targets police killings of black men, has led demands for reparations. The City of Chicago has paid out more than $50 million dollars in restitution, and issued a formal apology.

8. National Institute of Corrections (2013). Available at: http://nicic.gov/statestats/?st=IL (accessed 14 May 2015).

9. In part, this can be explained by the far higher availability of firearms in the United States. In Scotland, in the aftermath of a school shooting, the Firearms (Amendment) (No. 2) Act 1997 banned the private ownership of all handguns. However, it is also important to recognize the role of cultural history. Bladed weapons rather than firearms have always been embedded in Scottish culture. The national dress of Scotland, the kilt, includes a secreted dagger—the sgian dubh—in the right sock.

10. In the early part of the 20th century, Glasgow gained a reputation for working-class solidarity and ‘Red Clydeside’ socialist politics that set a tone for the city’s cultural politics that remains fundamental. During World War One, tenants associations formed common cause with new migrants to oppose increases in rent during war time. Led by women’s tenants groups, 30,000 residents went on rent strike, successfully persuading the council to freeze rents for the duration of the war. In the immediate post-war period, British Army tanks were called into Glasgow city centre to suppress an anticipated uprising.

11. It should also be noted that Richard J Daley, later mayor of Chicago for almost 20 years was a member of the Hamburg Athletic Association, a gang named by the Commission as one those responsible for the violence (Hagedorn, 2008).
